# The impact of virus infections on pneumonia mortality is complex in adults: a prospective multicentre observational study

**DOI:** 10.1186/s12879-017-2858-y

**Published:** 2017-12-06

**Authors:** Naoko Katsurada, Motoi Suzuki, Masahiro Aoshima, Makito Yaegashi, Tomoko Ishifuji, Norichika Asoh, Naohisa Hamashige, Masahiko Abe, Koya Ariyoshi, Konosuke Morimoto, on behalf of the Adult Pneumonia Study Group-Japan, Takao Wakabayashi, Naoto Hosokawa, Norihiro Kaneko, Kei Nakashima, Yoshihito Otsuka, Eiichiro Sando, Kaori Shibui, Daisuke Suzuki, Kenzo Tanaka, Kentaro Tochitani, Masayuki Chikamori, Masayuki Ishida, Hiroshi Nakaoka, Hiroyuki Ito, Kei Matsuki, Yoshiko Tsuchihashi, Bhim G. Dhoubhadel, Akitsugu Furumoto, Sugihiro Hamaguchi, Shungo Katoh, Satoshi Kakiuchi, Emi Kitashoji, Takaharu Shimazaki, Masahiro Takaki, Kiwao Watanabe, Lay-Myint Yoshida

**Affiliations:** 1 Department of Pulmonology, Kameda Medical Center, 929 Higashi-cho, Kamogawa, Chiba, Japan; 2 Division of Respiratory Medicine, Department of Internal Medicine, Kobe University Graduate School of Medicine, 7-5-1 Kusunoki-cho, Chuo-ku, Kobe, Japan; 3 Department of Clinical Medicine, Institute of Tropical Medicine, Nagasaki University, 1-12-4 Sakamoto, Nagasaki, 852-8523 Japan; 4 Department of General Internal Medicine, Kameda Medical Center, 929 Higashi-cho, Kamogawa, Chiba, Japan; 5 Department of Internal Medicine, Juzenkai Hospital, 7-18 Kagomachi, Nagasaki, Japan; 6 Department of Internal Medicine, Chikamori Hospital, 1-1-16 Okawasuji, Kochi, Japan; 7 Department of General Internal Medicine, Ebetsu City Hospital, 6 Wakakusacho, Ebetsu, Hokkaido Japan

**Keywords:** Pneumonia mortality, Chronic respiratory disease, Respiratory syncytial virus, Respiratory virus, Influenza, Paramyxovirus

## Abstract

**Background:**

Various viruses are known to be associated with pneumonia. However, the impact of viral infections on adult pneumonia mortality remains unclear. This study aimed to clarify the effect of virus infection on pneumonia mortality among adults stratified by virus type and patient comorbidities.

**Methods:**

This multicentre prospective study enrolled pneumonia patients aged ≥15 years from September 2011 to August 2014. Sputum samples were tested by in-house multiplex polymerase chain reaction assays to identify 13 respiratory viruses. Viral infection status and its effect on in-hospital mortality were examined by age group and comorbidity status.

**Results:**

A total of 2617 patients were enrolled in the study and 77.8% was aged ≥65 years. 574 (21.9%) did not have comorbidities, 790 (30.2%) had chronic respiratory disease, and 1253 (47.9%) had other comorbidities. Viruses were detected in 605 (23.1%) patients. Human rhinovirus (9.8%) was the most frequently identified virus, followed by influenza A (3.9%) and respiratory syncytial virus (3.9%). Respiratory syncytial virus was more frequently identified in patients with chronic respiratory disease (4.7%) than those with other comorbidities (4.2%) and without comorbidities (2.1%) (*p* = 0.037). The frequencies of other viruses were almost identical between the three groups. Virus detection overall was not associated with increased mortality (adjusted risk ratio (ARR) 0.76, 95% CI 0.53–1.09). However, influenza virus A and B were associated with three-fold higher mortality in patients with chronic respiratory disease but not with other comorbidities (ARR 3.38, 95% CI 1.54–7.42). Intriguingly, paramyxoviruses were associated with dramatically lower mortality in patients with other comorbidities (ARR 0.10, 95% CI 0.01–0.70) but not with chronic respiratory disease. These effects were not affected by age group.

**Conclusions:**

The impact of virus infections on pneumonia mortality varies by virus type and comorbidity status in adults.

**Electronic supplementary material:**

The online version of this article (10.1186/s12879-017-2858-y) contains supplementary material, which is available to authorized users.

## Background

Pneumonia is the major cause of morbidity and mortality among adults, especially in the elderly. Management of pneumonia is a critical problem in an ageing society like Japan. *Streptococcus pneumoniae* and *Haemophilus influenzae* are the leading bacterial causes of adult pneumonia, while viruses also play important roles in disease development. Recent advances in molecular diagnostic techniques have enabled us to detect multiple viruses simultaneously [1]. Studies have shown that viral infection is common in pneumonia patients [2, 3]. According to a recent systematic review and meta-analysis, viruses were detected in 24.5% of respiratory samples from community-acquired pneumonia (CAP) patients [4].

Various viruses are known to be associated with respiratory infections, including pneumonia. According to the systematic review, influenza is the most commonly detected virus in CAP, followed by human rhinovirus (HRV), respiratory syncytial virus (RSV), and human coronavirus (HCoV) [4]. In addition to these endemic respiratory viruses, emerging respiratory viruses, such as severe acute respiratory syndrome coronavirus, Middle East respiratory syndrome coronavirus, and avian influenza, are posing a particularly serious threat to global health security [5]. Studies have suggested that these emerging viral infections are associated with an increased risk of severe conditions and mortality among pneumonia patients [5, 6]. It must be noted that pneumonia mortality varies substantially according to patient characteristics, such as comorbidities, aspiration risk factors, and physical functional status [7, 8]. To establish effective control measures, high-priority viruses and patient groups must be identified. However, the prevalence of viruses in adult pneumonia and their virus-specific effects on clinical outcome remain largely unknown. To the best of our knowledge, no large-scale study has investigated the different effects of viruses on pneumonia mortality by patient characteristics.

We conducted this prospective multicentre study to determine the distribution of viruses associated with pneumonia in adults and to establish their virus-specific effects on pneumonia mortality stratified by age group and comorbidity status.

## Methods

### Study design, patient enrolment, and data collection

The Adult Pneumonia Study Group-Japan (APSG-J) conducted multicentre prospective hospital-based surveillance for community-onset pneumonia at four community-based hospitals in Japan. In our previous paper, the burden and aetiology of adult pneumonia were reported based on the data and clinical samples collected during the 1st phase of the study (September 2011 to January 2013) [9]. The current study included all data and samples collected during the whole study period (September 2011 to August 2014). Details of the study settings and enrolment criteria were described previously [9]. In brief, all outpatients and inpatients were screened by hospital physicians, and eligible patients were identified using a standardized case definition: patients aged ≥15 years with respiratory symptoms compatible with pneumonia and new infiltrative shadows on chest X-rays or computed tomography scans. Clinical information was collected from patients and medical charts using a standardized data collection form.

### Microbiological test

Sputum, blood, and urine samples were collected at the time of diagnosis. Gram staining, sputum culture, and blood culture were performed on site. Sputum samples were further tested by in-house multiplex polymerase chain reaction (PCR) assays to identify viral and bacterial pathogens at the Institute of Tropical Medicine, Nagasaki University. Thirteen viral pathogens (influenza A virus, influenza B virus, RSV, human metapneumovirus [hMPV], human parainfluenza virus [HPIV] type 1–4, HRV, HCoV 229E/OC43, human adenovirus [HAdV], and human bocavirus [HBoV]) and six bacterial pathogens (*Streptococcus pneumoniae*, *Haemophilus influenzae*, *Moraxella Catarrhalis*, *Mycoplasma pneumoniae*, *Chlamydophila pneumoniae*, and *Legionella pneumophila*) were tested using multiplex PCR assays. Details about the primers and PCR methods used have been described previously [10, 11]. Urinary antigen testing was performed for the detection of *S. pneumoniae* and *L. pneumophila* (Binax NOW *Streptococcus Pneumoniae*, Binax NOW *Legionella*; Alere Inc., Waltham, MA, USA).

### Definitions of variables

Diagnosis of viral infection was made according to PCR results. Bacterial infection was diagnosed when any of the following criteria were fulfilled: 1) culture yielded pathogenic bacteria from microscopically purulent sputum samples (i.e., Geckler’s classification groups 4 and 5) or normally sterile site samples; 2) PCR assays were positive for bacterial DNA in microscopically purulent sputum samples; or 3) urinary antigen tests showed a positive result.

Patients were categorized into four age groups: 15–64 years, 65–74 years, 75–84 years, and ≥85 years. Patients’ disability status was evaluated using the Eastern Cooperative Oncology Group Performance Status (PS) score [12]. Pneumonia severity was assessed using the CURB65 scoring system [13]. To estimate the effect on pneumonia mortality, viruses were categorized into four groups: 1) HRV; 2) influenza A and B viruses; 3) paramyxoviruses (RSV, hMPV, and PIV type 1–4); and 4) other viruses (HAdV, HBoV, and HCoV).

We divided patients into three groups according to comorbidity status: 1) patients without comorbidity; 2) patients with chronic respiratory disease; and 3) patients with comorbidities other than chronic respiratory disease (i.e., other comorbidities). Chronic respiratory disease included bronchial asthma, chronic obstructive pulmonary disease (COPD), interstitial pneumonia, pneumoconiosis, and bronchiectasis. Other comorbidities included diabetes mellitus, cerebrovascular disease, dementia, neuromuscular disease, cardiac failure, ischaemic heart disease, collagen disease, malignancy, renal disease, and liver disease. Patients were considered to have aspiration risk factors when they had any of the following factors: episodes of aspiration, the presence of dysphagia, consciousness disturbances, neuromuscular diseases, cerebrovascular diseases, tube feeding, and bedridden status [14].

The in-hospital death was defined as any death occurred during the hospitalization. During the first year of study, we followed up our patients after the enrolment and confirmed that no outpatient had died within 30 days of enrolment. We therefore considered the in-hospital death as a good marker of short-term mortality in pneumonia patients regardless of their hospitalization status.

### Statistical analysis

Patients were categorized according to their comorbidity status (i.e., patients without comorbidity, with chronic respiratory disease, or with other comorbidities) and compared using chi-squared tests. Viral and bacterial infection status were compared by age group and comorbidity status using chi-squared tests, Fisher’s exact tests, and chi-squared tests for trend. In-hospital mortality rates were calculated by viral and bacterial infection status and compared with those of the virus-negative group. The effects of viral infection on in-hospital mortality were expressed as risk ratios with 95% confidence intervals (CI) and estimated using Poisson regression models with robust standard errors. Age, study site, comorbidity status, duration of symptoms, month of diagnosis, antibiotic use, and presence of bacteria were considered potential confounders based on prior knowledge and were included in the multiple regression models. For patients whose onset of symptoms were unknown (<5%), we coded those missing values as “unknown” and included all patients in our analysis. The data were analysed using STATA version 13 (STATA Corp., College Station, TX, USA).

### Ethics

This study was approved by the institutional review boards (IRBs) of the Institute of Tropical Medicine, Nagasaki University, Ebetsu City Hospital, Kameda Medical Center, Chikamori Hospital, and Juzenkai Hospital. The requirement for obtaining written consent from all participants was waived by all IRBs because of the study’s observational nature without any deviation from the current medical practice. Anonymized data were used for the analyses.

## Results

### Clinical characteristics

**Fig. 1:**
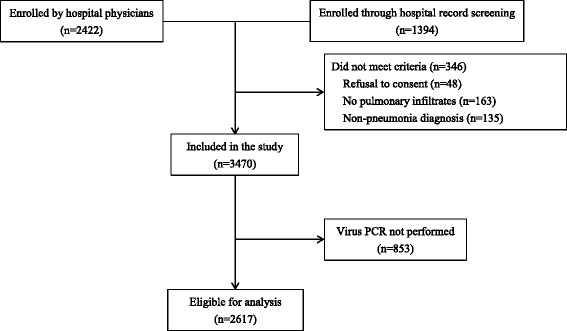
Flow chart of study patients

During the study period, 3816 patients were enrolled in the study. Of these, 346 were excluded because of refusal to participate in the study (*n* = 48), absence of pulmonary infiltrates (*n* = 163), and non-pneumonia diagnosis (*n* = 135). After excluding 853 (22.3%) patients whose sputum samples for PCR assays were unavailable, 2617 patients were eligible for analysis (Fig. 1).

Table 1 shows the clinical characteristics of pneumonia patients by comorbidity status. Approximately 60% of patients were male, and the median age was 73.8 years (interquartile range, 66 to 85 years). The proportions of patients aged ≥65 years and ≥85 years were 77.8 and 28.4%, respectively; 46.7% of 2545 patients had aspiration risk factors. Of all patients, 574 (21.9%) did not have comorbidities, 790 (30.2%) had chronic respiratory disease, and 1253 (47.9%) had other comorbidities. Patients with comorbidities were more likely to be male, older, more frequently required hospitalization, more frequently developed severe disease, more frequently had aspiration risk factors, had higher PS scores, and visited the hospital earlier than those without comorbidities. The proportion of patients with aspiration risk factors was particularly high (65.5%) among patients with other comorbidities. Multiple symptoms were most frequently observed in patients with chronic respiratory disease (70.1%), followed by those without comorbidities (67.9%) and those with other comorbidities (57.5%).

**Table 1:** Characteristics of enrolled pneumonia patients

	Total	No comorbidity	Chronic respiratory disease	Other comorbidities^a^	*P* value†
	*N* = 2617	*N* = 574	*N* = 790	*N* = 1253	
	N (%)	N (%)	N (%)	N (%)	
Male sex	1591 (60.8)	295 (51.4)	540 (68.4)	756 (60.3)	<0.001
Age group					
15–64	580 (22.2)	268 (46.7)	121 (15.3)	191 (15.2)	<0.001
65–74	447 (17.1)	91 (15.9)	141 (17.9)	215 (17.2)	
75–84	848 (32.4)	126 (22.0)	321 (40.6)	401 (32.0)	
85+	742 (28.4)	89 (15.5)	207 (26.2)	446 (35.6)	
Admission	2007 (76.7)	305 (53.1)	597 (75.6)	1105 (88.2)	<0.001
Performance status ≥3, *n* = 1307	310 (23.7)	37 (11.1)	56 (13.9)	217 (37.9)	<0.001
Severe disease (CURB65 ≥ 4), *n* = 2100	154 (7.3)	19 (4.5)	41 (6.7)	94 (8.9)	0.010
Duration of symptoms^b^ > 3 days, *n* = 2507	874 (34.9)	256 (47.8)	251 (33.3)	367 (30.2)	<0.001
No. of symptoms ≥3	1664 (63.6)	390 (67.9)	554 (70.1)	720 (57.5)	<0.001
Aspiration risk, *n* = 2545	1189 (46.7)	103 (18.4)	293 (37.8)	793 (65.5)	<0.001
Period					
Spring (January to March)	645 (24.7)	129 (22.5)	195 (24.7)	321 (25.6)	0.092
Summer (April to June)	572 (21.9)	147 (25.6)	161 (20.4)	264 (21.1)	
Autumn (July to September)	641 (24.5)	151 (26.3)	189 (23.9)	301 (24.0)	
Winter (October to December)	759 (29.0)	147 (25.6)	245 (31.0)	367 (29.3)	
Study site					
Ebetsu	326 (12.4)	79 (13.8)	123 (15.6)	124 (9.9)	<0.001
Kamogawa	1379 (52.7)	348 (60.6)	416 (52.7)	615 (49.1)	
Kochi	600 (22.9)	96 (16.7)	153 (19.4)	351 (28.0)	
Nagasaki	312 (11.9)	51 (8.9)	98 (12.4)	163 (13.0)	

†Chi-square tests were performed to compare three groups

^a^Other comorbidities include diabetes mellitus, cerebrovascular disease, dementia, neuromuscular disease, cardiac failure, ischaemic heart disease, collagen disease, malignancy, renal disease, and liver disease

^b^Symptoms include cough, sputum, chest pain, fever, chill, headache, fatigue, appetite loss, myalgia, arthralgia, nausea, vomiting, diarrhoea, and dehydration

### Detection of viral and bacterial pathogens

In total, 605 (23.1%) patients tested positive for at least one virus (Table 2). HRV was the most common virus identified (*n* = 256 [9.8%]), followed by influenza A (*n* = 101 [3.9%]) and RSV (*n* = 101 [3.9%]). Two or more viruses were detected in 31 patients (1.2%). The most frequent combinations of viruses were HRV plus influenza A (*n* = 7), followed by HRV plus RSV (*n* = 4), and HRV plus hMPV (*n* = 4). Three viruses (HRV, hMPV, and PIV type 3) were detected in one patient. Bacterial pathogens were detected in 992 (37.9%) patients, and both viral and bacterial pathogens were detected in 246 (9.4%) patients (i.e., viral-bacterial co-infection).

**Table 2:** Viral and bacterial infection status among pneumonia patients by age group

	Total	15–64 y	65–74 y	75–84 y	85 y+	*P* value*
Viruses	*N* = 2617	*N* = 580	*N* = 447	*N* = 848	*N* = 742	
HRV	256 (9.8)	70 (12.1)	42 (9.4)	69 (8.1)	75 (10.1)	0.185
Inf A	101 (3.9)	22 (3.8)	13 (2.9)	32 (3.8)	34 (4.6)	0.343
RSV	101 (3.9)	12 (2.1)	20 (4.5)	32 (3.8)	37 (5.0)	0.016
PIV 3	49 (1.9)	14 (2.4)	7 (1.6)	17 (2.0)	11 (1.5)	0.311
HMPV	45 (1.7)	4 (0.7)	13 (2.9)	14 (1.7)	14 (1.9)	0.286
PIV 1	26 (2.0)	5 (0.9)	3 (0.7)	10 (1.2)	8 (1.1)	0.523
Inf B	24 (0.9)	6 (1.0)	4 (0.9)	3 (0.4)	11 (1.5)	0.588
PIV 2	14 (0.5)	4 (0.7)	3 (0.7)	3 (0.4)	4 (0.5)	0.567
HCoV (229E/OC43)	13 (0.5)	6 (1.0)	3 (0.7)	2 (0.2)	2 (0.3)	0.029
HAdV	6 (0.2)	1 (0.2)	1 (0.2)	4 (0.5)	0 (0.0)	0.708
HBoV	2 (0.1)	1 (0.2)	0 (0.0)	1 (0.1)	0 (0.0)	0.394
PIV 4	0 (0.0)	0 (0.0)	0 (0.0)	0 (0.0)	0 (0.0)	NA
Any viruses	605 (23.1)	137 (23.6)	102 (22.8)	180 (21.2)	186 (25.1)	0.677
≥2 viruses	31 (1.2)	8 (1.4)	7 (1.6)	6 (0.7)	10 (1.3)	0.654
Any bacterial pathogens	992 (37.9)	265 (45.7)	194 (43.4)	288 (34.0)	245 (33.0)	<0.001
Viral-bacterial co-infection	246 (9.4)	66 (11.4)	46 (10.3)	69 (8.1)	65 (8.8)	0.056
Any viral and bacterial pathogens	1351 (51.6)	336 (57.9)	250 (55.9)	399 (47.1)	366 (49.3)	<0.001

*HRV* human rhinovirus, *InfA* influenza A virus, *RSV* respiratory syncytial virus, *PIV1–4* human parainfluenza virus type 1–4, *HMPV* human metapneumovirus, *InfB* influenza B virus, *HCoV* human coronavirus (229E/OC43), *HAdV* human adenovirus, *HBoV* human bocavirus

*Chi-square tests for trend were performed

Viral and bacterial infection status were compared by age group (Table 2) and comorbidity status (Table 3). The proportion of overall virus-positive pneumonia did not differ by age group. RSV was more frequently identified in older age groups, while HCoV was more frequently identified in younger age groups. The proportion of influenza-positive pneumonia was similar across all age groups. Bacterial pathogens were more frequently identified in younger patients. For patients’ comorbidity status, RSV was most frequently identified in patients with chronic respiratory disease (4.7%), followed by those with other comorbidities (4.2%) and without comorbidities (2.1%) (*p* = 0.037); the frequencies of other viruses were almost identical between the three groups (Table 3). Bacterial pathogens were more frequently identified in patients without comorbidities than in those with comorbidities.

**Table 3:** Viral and bacterial infection status among pneumonia patients by comorbidity status

	No comorbidities	Chronic respiratory disease	Other comorbidities^a^	*P* value†
Viruses	*N* = 574	*N* = 790	*N* = 1253	
HRV	57 (9.9)	87 (11.0)	112 (8.9)	0.304
Inf A	21 (3.7)	29 (3.7)	51 (4.1)	0.866
RSV	12 (2.1)	37 (4.7)	52 (4.2)	0.037
PIV 3	12 (2.1)	11 (1.4)	26 (2.1)	0.492
HMPV	6 (1.1)	13 (1.7)	26 (2.1)	0.286
PIV 1	4 (0.7)	10 (1.3)	12 (1.0)	0.598
Inf B	5 (0.9)	5 (0.6)	14 (1.1)	0.596
PIV 2	3 (0.5)	4 (0.5)	7 (0.6)	1.000
HCoV (229E/OC43)	3 (0.5)	5 (0.6)	5 (0.4)	0.767
HAdV	2 (0.4)	1 (0.1)	3 (0.2)	0.764
HBoV	0 (0.0)	0 (0.0)	2 (0.2)	0.711
PIV 4	0 (0.0)	0 (0.0)	0 (0.0)	NA
Any viruses	118 (20.6)	196 (24.8)	291 (23.2)	0.183
≥2 viruses	7 (1.2)	6 (0.8)	18 (1.4)	0.386
Any bacterial pathogens	233 (40.6)	315 (39.9)	444 (35.4)	0.043
Viral-bacterial co-infection	54 (9.4)	88 (11.1)	104 (8.3)	0.101
Any viral and bacterial pathogens	297 (51.7)	423 (53.5)	631 (50.4)	0.373

*HRV* human rhinovirus, *InfA* influenza A virus, *RSV* respiratory syncytial virus, *PIV1–4* human parainfluenza virus type 1–4, *HMPV* human metapneumovirus, *InfB* influenza B virus, *HCoV* human coronavirus (229E/OC43), *HAdV* human adenovirus, *HBoV* human bocavirus

† Chi-square tests or Fisher’s exact tests were performed to compare the three groups

^a^Other comorbidities include diabetes mellitus, cerebrovascular disease, dementia, neuromuscular disease, cardiac failure, ischaemic heart disease, collagen disease, malignancy, renal disease, and liver disease

We explored symptoms of patients with each respiratory virus groups (Additional file 1: Table S1). The proportion of patients with multiple symptoms (i.e., the number of symptoms ≥3) was higher in patients with paramyxovirus infection than those without viral infection (75.0% vs 61.3%, *p* < 0.001). In the group of patients with aspiration risk factors, those with paramyxovirus were more likely to have a cough than patients without virus (71.1% vs 46.2%, *p* < 0.001).

### In-hospital mortality of pneumonia and virus detection

Among 2617 patients, 193 patients died before discharge, with an overall in-hospital mortality of 7.4%. The mortalities among virus-positive and -negative groups were 5.8 and 7.9%, respectively, and the overall effect (adjusted risk ratio [ARR]) of viruses on mortality was 0.76 (95% CI 0.53–1.09, *p* = 0.140). Intriguingly, when the effect of specific virus type was analysed, paramyxoviruses, including RSV, hMPV, and PIV type 1–4, were associated with a dramatically lower mortality (ARR 0.29, 95% CI 0.12–0.71, *p* = 0.007). Among 212 paramyxovirus-positive pneumonia, five died: three were RSV-positive, one was HMPV-positive, and one was HMPV-positive at the enrolment. RSV alone was also associated with a lower mortality (ARR 0.48, 95% CI 0.18–1.29, *p* = 0.146), but the association did not reach a statistically significant level. None of the other virus types were associated with mortality.

The virus type-specific effects were further investigated after patients were stratified by age group and comorbidity status (Tables 4 and 5). Similar effects of viruses were seen across all age groups. However, influenza virus A and B were strongly associated with higher mortality in patients with chronic respiratory disease (ARR 3.38, 95% CI 1.54–7.42, *p* = 0.002), while no influenza-related death was observed in those without comorbidity. Intriguingly, paramyxoviruses were associated with markedly lower mortality in patients with other comorbidities (ARR 0.10, 95% CI 0.01–0.70, *p* = 0.020), but this association was not observed in other groups. HRV was not associated with mortality in the three groups. Virus only, bacteria only, and both virus- and bacteria-positive pneumonia demonstrated higher mortality than virus- and bacteria-negative pneumonia in patients with chronic respiratory disease, but these associations did not reach statistically significant levels.

**Table 4:** Viral and bacterial infection status and in-hospital mortality among pneumonia patients by age group

	Total, *n* = 2617		15–64 y, *n* = 580		65–74 y, *n* = 447		75–84 y, *n* = 848		85 y+, *n* = 742	
	No. death/no. cases (% mortality)	ARR^a^ (95% CI)	No. death/no. cases (% mortality)	ARR^a^ (95% CI)	No. death/no. cases (% mortality)	ARR^a^ (95% CI)	No. death/no. cases (% mortality)	ARR^a^ (95% CI)	No. death/no. cases (% mortality)	ARR^a^ (95% CI)
HRV	14/234 (6.0)	0.86 (0.51–1.45)	2/63 (3.2)	1.60 (0.38–6.65)	3/37 (8.1)	1.16 (0.38–3.59)	3/66 (4.6)	0.55 (0.17–1.74)	6/68 (8.8)	0.87 (0.40–1.93)
Inf A/B	10/110 (9.1)	1.13 (0.60–2.13)	1/25 (4.0)	1.74 (0.17–17.74)	1/15 (6.7)	1.03 (0.12–8.86)	3/31 (9.7)	0.93 (0.29–2.99)	5/39 (12.8)	1.44 (0.58–3.61)
Paramyxovirus (RSV/hMPV/PIV1–4)	5/212 (2.4)	0.29 (0.12–0.71)	0/35 (0.0)	0.00 (0.00–0.00)	1/40 (2.5)	0.41 (0.06–3.01)	3/70 (4.3)	0.44 (0.14–1.39)	1/67 (1.5)	0.15 (0.02–1.05)
Other viruses (HAdV/HBoV/HCoV)	2/18 (11.1)	1.62 (0.42–6.18)	1/6 (16.7)	4.30 (0.63–29.53)	0/3 (0.0)	0.00 (0.00–0.00)	1/7 (14.3)	1.66 (0.26–10.64)	0/2 (0.0)	0.00 (0.00–0.00)
Multiple viruses	4/31 (12.9)	1.69 (0.67–4.27)	0/8 (0.0)	0.00 (0.00–0.00)	1/7 (14.3)	2.63 (0.37–18.75)	0/6 (0.0)	0.00 (0.00–0.00)	3/10 (30.0)	2.72 (0.88–8.46)
No virus	158/2012 (7.9)	Reference	15/443 (3.4)	Reference	25/345 (7.3)	Reference	62/668 (9.3)	Reference	56/556 (10.1)	Reference
		ARR^b^(95% CI)		ARR^b^(95% CI)		ARR^b^(95% CI)		ARR^b^(95% CI)		ARR^b^(95% CI)
Only viruses	19/359 (5.3)	0.66 (0.41–1.07)	2/71 (2.8)	0.96 (0.18–5.02)	1/56 (1.8)	0.27 (0.03–2.20)	7/111 (6.3)	0.70 (0.32–1.56)	9/121 (7.4)	0.68 (0.34–1.39)
Only bacterial pathogens	51/746 (6.8)	0.90 (0.65–1.25)	5/199 (2.5)	0.82 (0.28–2.42)	10/148 (6.8)	1.02 (0.48–2.17)	21/219 (9.6)	1.05 (0.64–1.74)	15/180 (8.3)	0.76 (0.43–1.35)
Viral-bacterial co-infection	16/246 (6.5)	0.85 (0.51–1.41)	2/66 (3.0)	1.31 (0.25–6.80)	5/46 (10.9)	1.75 (0.67–4.60)	3/69 (4.4)	0.46 (0.14–1.49)	6/65 (9.2)	0.85 (0.37–1.94)
No viral or bacterial pathogens	107/1266 (8.5)	Reference	10/244 (4.1)	Reference	15/197 (7.6)	Reference	41/449 (9.1)	Reference	41/376 (10.9)	Reference

*ARR* adjusted risk ratio, *CI* confidence interval, *HRV* human rhinovirus, *InfA* influenza A virus, *RSV* respiratory syncytial virus, *PIV1–4* human parainfluenza virus type 1–4, *HMPV* human metapneumovirus, *InfB* influenza B virus, *HCoV* human coronavirus (229E/OC43), *HAdV* human adenovirus, *HBoV* human bocavirus

^a^Adjusted for age, study site, comorbidity status, duration of symptoms, month of diagnosis, antibiotic use, and presence of bacteria

^b^Adjusted for age, study site, comorbidity status, duration of symptoms, month of diagnosis, and antibiotic use

**Table 5:** Viral and bacterial infection status and in-hospital mortality among pneumonia patients by comorbidity status

	Without comorbidities, *n* = 574		With chronic respiratory disease, *n* = 790		With other comorbidities^a^, *n* = 1253	
	No. death/no. cases (% mortality)	ARR^b^ (95% CI)	No. death/no. cases (% mortality)	ARR^b^ (95% CI)	No. death/no. cases (% mortality)	ARR^b^ (95% CI)
HRV	2/53 (3.8)	0.73 (0.18–2.96)	4/83 (4.8)	0.78 (0.28–2.14)	8/98 (8.2)	0.97 (0.48–1.96)
Inf A/B	0/22 (0.0)	0.00 (0.00–0.00)	6/31 (19.4)	3.38 (1.54–7.42)	4/57 (7.0)	0.73 (0.26–2.02)
Paramyxovirus (RSV/hMPV/PIV1–4)	1/32 (3.1)	0.47 (0.07–3.26)	3/71 (4.2)	0.66 (0.20–2.13)	1/109 (0.9)	0.10 (0.01–0.70)
Other viruses (HAdV/HBoV/HCoV)	0/4 (0.0)	0.00 (0.00–0.00)	1/5 (20.0)	4.55 (0.58–35.5)	1/9 (11.1)	1.33 (0.21–8.66)
Multiple viruses	0/7 (0.0)	0.00 (0.00–0.00)	1/6 (16.7)	3.98 (0.68–23.24)	3/18 (16.7)	1.68 (0.56–5.03)
No virus	26/456 (5.7)	Reference	44/594 (7.4)	Reference	88/962 (9.2)	Reference
		ARR^c^(95% CI)		ARR^c^(95% CI)		ARR^c^(95% CI)
Only viruses	1/64 (1.6)	0.24 (0.03–1.78)	9/108 (8.3)	1.28 (0.59–2.81)	9/187 (4.8)	0.51 (0.26–1.01)
Only bacterial pathogens	8/179 (4.5)	0.83 (0.36–1.93)	16/227 (7.1)	1.13 (0.61–2.09)	27/340 (7.9)	0.84 (0.54–1.31)
Viral-bacterial co-infection	2/54 (3.7)	0.58 (0.14–2.38)	6/88 (6.8)	1.29 (0.55–3.06)	8/104 (7.7)	0.77 (0.38–1.59)
No viral or bacterial pathogens	18/277 (6.5)	Reference	28/367 (7.6)	Reference	61/622 (9.8)	Reference
		ARR^b^ (95% CI)		ARR^b^ (95% CI)		ARR^b^ (95% CI)
Multiple viruses	0/7 (0.0)	0.00 (0.00–0.00)	1/6 (16.7)	3.22 (0.52–19.81)	3/18 (16.7)	2.98 (0.91–9.78)
Single virus	3/111 (2.7)	Reference	14/190 (7.4)	Reference	14/273 (5.1)	Reference

*ARR* adjusted risk ratio, *CI* confidence interval, *HRV* human rhinovirus, *InfA* influenza A virus, *RSV* respiratory syncytial virus, *PIV1–4* human parainfluenza virus type 1–4, *HMPV* human metapneumovirus, *InfB* influenza B virus, *HCoV* human coronavirus (229E/OC43), *HAdV* human adenovirus, *HBoV* human bocavirus

^a^ Other comorbidities include diabetes mellitus, cerebrovascular disease, dementia, neuromuscular disease, cardiac failure, ischaemic heart disease, collagen disease, malignancy, renal disease, and liver disease

^b^Adjusted for age, study site, duration of symptoms, month of diagnosis, antibiotic use and presence of bacteria

^c^Adjusted for age, study site, duration of symptoms, month of diagnosis, and antibiotic use

We explored the association between viruses and in-hospital mortality in patients with aspiration risk factors (Additional file 1: Table S2). Paramyxovirus was the only virus type significantly associated with reduced mortality in this category of patients (ARR 0.28, 95% CI 0.09–0.85, *p* = 0.024). Influenza A and B were not associated with mortality (ARR 1.78, 95% CI 0.92–3.47, *p* = 0.089). The mortality was higher among patients with cough than those without cough (11.7% vs 4.9%, *p* < 0.001).

## Discussion

In this multicentre prospective study, 23.1% of adult pneumonias were associated with viruses. HRV was the leading virus identified, followed by influenza A and RSV. This pattern was almost identical across all age groups. Influenza was strongly associated with higher mortality in patients with chronic respiratory disease but not in other groups. Paramyxoviruses, including RSV, hMPV, and PIV type 1–4, were associated with improved survival in patients with other comorbidities, especially in those with aspiration risk factors. To the best of our knowledge, this study is the first to systematically investigate virus-specific effects on pneumonia mortality by age group and comorbidity status among adults.

Viruses are frequently observed in pneumonia patients. According to previous studies, viruses were positive in 23, 34, 30 and 28% of CAP patents in the US [2], Norway [3], UK [15], and China [16], respectively, and HRV, influenza A, and RSV were the leading viruses identified; these findings were confirmed in our study. However, the role of viruses in pneumonia development and progression has not been fully established. A systematic review showed that the risk of death was higher in patients with viral infection, although the association did not reach a statistically significant level (odds ratio 1.3, 95% CI 0.8–2.2) [4]. The major limitation of previous studies is that all viruses and patient groups were pooled, which may have overlooked their intergroup differences. In fact, in the current study, viruses overall were not associated with increased mortality among all pneumonia patients (ARR 0.76, 95% CI 0.53–1.09), but the effects were different by viruses and patient characteristics.

Influenza increased pneumonia mortality by 3.4-fold (95% CI 1.54–7.42) in our patients with chronic respiratory disease but did not change the mortality in other patients. Although influenza is known to be an important cause of pneumonia and death, only a few studies have formally compared the mortality of influenza pneumonia with that of non-influenza pneumonia, and the findings have been inconsistent [17, 18]. On the other hand, previous studies have demonstrated that chronic respiratory disease increases the risk of severe outcome among influenza patients [19, 20]. Bronchial epithelial cells of COPD are susceptible to replication of influenza virus because of their impaired antiviral immunity [21]; thus, the effect of influenza on disease progression may be stronger in patients with this condition. According to the Cochrane review, influenza vaccination reduces exacerbations in patients with COPD [22]. Seasonal influenza vaccination campaigns must therefore pay special attention to this patient group.

Interestingly, paramyxoviruses including RSV were associated with improved survival in our patients with other comorbidities. Inconsistent findings have been reported about the effect of paramyxoviruses on pneumonia severity. A multinational study showed that older patients who had been infected with RSV were more likely to be hospitalized than those with other respiratory viruses [23], while a study conducted in the US demonstrated that patients with RSV infection were less frequently hospitalized than those with influenza infection [24]. A retrospective cohort study conducted in Hong Kong showed that the 30-day and 60-day mortality rates were similar between adult patients hospitalized with RSV and those with seasonal influenza [25]. These inconsistent findings suggest that the effects of paramyxovirus infection substantially vary by patients’ conditions. In fact, in the current study, compared with virus-negative pneumonia, the mortality of paramyxovirus-associated pneumonia was substantially lower among patients with other comorbidities but this finding was not observed among patients without comorbidities and patients with chronic respiratory disease. The low mortality of paramyxovirus-associated pneumonia in this groups may be associated with its high prevalence of multiple symptoms. In our study, the proportion of patients with multiple symptoms (i.e., the number of symptoms ≥3) was higher in patients with paramyxovirus infection than those without viral infection. Patients with paramyxovirus-associated pneumonia are more likely to develop symptoms and are probably more likely to visit hospitals, and this benefit may be observed in patients with comorbidities. In the group of patients with aspiration risk factors, those with paramyxovirus-associated pneumonia were more likely to have a cough than patients without virus (71.1% vs 46.2%, *p* < 0.001), and the mortality was higher among patients with cough than those without cough (11.7% vs 4.9%, *p* < 0.001). The low mortality of paramyxovirus-associated pneumonia in patients with aspiration risk factors also suggests that these viruses may stimulate the cough reflex and improve patients’ survival; however, our study does not provide conclusive evidence. Further studies are needed to unveil the mechanisms of potential benefits of paramyxovirus infection on pneumonia mortality.

In our study, multiple viruses were identified in 5.1% of virus-associated pneumonia and were associated with higher mortality than single viral infection in patients with chronic respiratory disease and other comorbidities. The association between multiple viral infections and pneumonia mortality remains uncertain [26]. Systematic reviews have shown that multiple viral infections in patients with respiratory disease are not associated with disease severity [27, 28]; however, the majority of previous studies included young children but not adults. The effect of multiple viruses on disease progression may be different in children and adults. Consistent with previous studies [3, 29, 30], half of the viral pneumonia patients were co-infected with bacterial pathogens. A systematic review showed that viral-bacterial co-infection increased mortality [4]; however, this association was not observed in our study. Viral infections of the lower respiratory tract may increase the risk of secondary bacterial infection, which may increase the risk of pneumonia and mortality [26]. However, sputum samples were collected once in our study; thus, we were unable to determine the time course of viral and bacterial infections. A cohort study with sequential respiratory sampling may be a preferable design to establish a causal association between viral-bacterial co-infection and pneumonia mortality.

Advances in molecular diagnostic techniques have enabled virus detection in clinical settings. Our findings raise the question of whether all pneumonia patients should be tested for viruses. The increased mortality of influenza-associated pneumonia in patients with chronic respiratory disease suggests the importance of early diagnosis of influenza and initiation of antivirals for this patient group. On the other hand, no substantial increase of mortality was found for other viruses. Screening for viruses in all pneumonia patients may be unnecessary in clinical settings.

Our study has limitations. First, sputum samples were not available for 25% of the enrolled patients. However, the clinical characteristics of patients without sputum samples did not differ from those of patients with sputum samples. Exclusion of this group did not affect our findings. Second, we used PCR to detect viruses. The detection of viral RNA and DNA in respiratory samples does not always indicate the presence of a causal pathogen; particularly, the detection of viruses in nasopharyngeal swabs may not be reflecting lower respiratory infections in pneumonia patients. We, therefore, used sputum samples from pneumonia patients, and the presence of viruses in the lower respiratory tract must be very likely causative [31]. Third, due to the nature of an observational study design, unmeasured confounding factors may have remained in our risk factor analyses for pneumonia mortality.

## Conclusions

Viral infections are common in adult pneumonia, and their impact on pneumonia mortality varies by viruses and comorbidities. Variable impacts of viruses by population characteristics must be considered in the development of antiviral drugs and vaccines.

## Additional file

Additional file 1: Table S1. Proportion of patients with multiple symptoms (number of symptoms ≥3) by virus. **Table S2.** Viral and bacterial infection status and in-hospital mortality among pneumonia patients with and without aspiration risk factors. (DOCX 17 kb)

## Abbreviations

ARR: Adjusted risk ratio
CAP: Community-acquired pneumonia
CI: Confidence intervals
COPD: Chronic obstructive pulmonary disease
HAdV: Human adenovirus
HBoV: Human bocavirus
HCoV: Human coronavirus
hMPV: Human metapneumovirus
HPIV: Human parainfluenza virus
HRV: Human rhinovirus
PCR: Polymerase chain reaction
PS: Performance Status
RSV: Respiratory syncytial virus
