# Supplementary material for: The impact of virus infections on pneumonia mortality is complex in adults: a prospective multicentre observational study
Source: BMC Infect Dis. 2017 Dec 6;17:755. doi: 10.1186/s12879-017-2858-y (PMC5719746; doi:10.1186/s12879-017-2858-y)
Supplement: Additional file 1: Table S1. — Proportion of patients with multiple symptoms (number of symptoms ≥3) by virus. Table S2. Viral and bacterial infection status and in-hospital mortality among pneumonia patients with and without aspiration risk factors. (DOCX 17 kb) [file 12879_2017_2858_MOESM1_ESM.docx]

**Additional file 1**

**Table S1.** Proportion of patients with multiple symptoms (number of symptoms ≥3) by virus.

| Viruses | No. of patients with multiple symptoms/no. of total patients (%) |
| --- | --- |
| HRV | 166/234 (70.9) |
| Inf A/B | 74/110 (67.3) |
| Paramyxovirus (RSV/hMPV/PIV1-4) | 159/212 (75.0) |
| Other virus (HAdV/HBoV/HCoV) | 10/18 (55.6) |
| Multiple viruses | 22/31 (71.0) |
| No virus | 1,233/2012 (61.3) |

**Table S2.** Viral and bacterial infection status and in-hospital mortality among pneumonia patients with and without aspiration risk factors.

|  | Without aspiration risk factors | With aspiration risk factors |
| --- | --- | --- |
|  | n=1356 | n=1189 |
|  | ARR* (95% CI) | ARR* (95% CI) |
| HRV | 0.96 (0.35-2.46) | 0.79 (0.41-1.50) |
| Inf A/B | 0.36 (0.05-2.68) | 1.78 (0.92-3.47) |
| Paramyxovirus (RSV/hMPV/PIV1-4) | 0.35 (0.08-1.49) | 0.28 (0.09-0.85) |
| Other virus (HAdV/HBoV/HCoV) | 0.00 (0.00-0.00) | 2.21 (0.64-7.62) |
| Multiple viruses | 1.53 (0.19-12.64) | 1.86 (0.68-5.05) |
| No virus | Reference | Reference |
|  |  |  |
|  | ARR† (95% CI) | ARR† (95% CI) |
| Only viruses | 0.45 (0.15-1.31) | 0.78 (0.45-1.33) |
| Only bacterial pathogens | 0.72 (0.39-1.31) | 1.07 (0.72-1.58) |
| Viral and bacterial co-infection | 0.62 (0.22-1.71) | 1.09 (0.60-1.99) |
| No viral or bacterial pathogen | Reference | Reference |

ARR=adjusted risk ratio, CI=confidence interval, HRV=human rhinovirus, InfA=influenza A virus, RSV=respiratory syncytial virus, PIV1-4=human parainfluenza virus type 1-4, HMPV=human metapneumovirus, InfB=influenza B virus, HCoV=human coronavirus (229E/OC43), HAdV=human adenovirus, HBoV=human bocavirus.

* Adjusted for age, study site, comorbidity status, duration of symptoms, month of diagnosis, antibiotics use and presence of bacteria.

†Adjusted for age, study site, comorbidity status, duration of symptoms, month of diagnosis, and antibiotics use.
